# Endocarditis Caused by Gram-Negative *Moraxella osloensis* in an Immunocompetent Patient: First Case Report in Latin America

**DOI:** 10.1155/2018/4209094

**Published:** 2018-06-06

**Authors:** Patricia Fraga Paiva, Carolina Fraga Paiva, Emanoel Guimarães Paiva, Gisele Maria Campos Fabri, José Fabri Junior

**Affiliations:** ^1^Medicine School, Faculty of Medical Sciences and Health of Juiz de Fora Suprema and Therezinha de Jesus Hospital and Maternity, 33 Dirceu de Andrade St., 36025-140 Juiz de Fora, MG, Brazil; ^2^Nursing School, Faculty of Medical Sciences and Health of Juiz de Fora Suprema and Therezinha de Jesus Hospital and Maternity, 33 Dirceu de Andrade St., 36025-140 Juiz de Fora, MG, Brazil; ^3^Intensive Care and Emergency Rooms, Santa Casa de Misericórdia Hospital, 3353 Barão do Rio Branco Ave, 36021-630 Juiz de Fora, MG, Brazil; ^4^Dentistry School, Juiz de Fora Federal University, José Lourenço Kelmer St., s/n Martelos, 36036-330 Juiz de Fora, MG, Brazil

## Abstract

This is a case report of infective endocarditis due to *Moraxella osloensis*. This study would be the fourth since the two latest episodes were described in 2015. The patient of this exposition is different from those previously described in the literature because it was a young adult, under 50 years of age, immunocompetent, with no comorbidities and no obvious focus of infection.

## 1. Introduction

The genus *Moraxella* consists of aerobic gram-negative coccobacilli, and it was first described in 1967. However, few rare cases have been reported in the literature since then [1, 2]. The natural habitat of *Moraxella osloensis* and its clinical significance in infections are much less studied due to rare individual reports. However, it has been observed in healthy adults' nasopharynx [3, 4], and in outpatients' ear, nose [5] and throat [1]. The infections caused by *M. osloensis* include infective endocarditis (IE), meningitis, osteomyelitis, septic arthritis, vaginitis, subclinical sinusitis [6] and bacteremia [7], which does not usually occur alone.

## 2. Case Report

The 41-year-old male patient F.C.F. showed no comorbidities and denied alcoholism, smoking, and taking regular medication. He started having fever, myalgia, anorexia, vomiting, jaundice, and colure on July 14, 2016. He took antiviral drugs with monohydrate dipyrone (750 mg/day) associated to chlorpheniramine maleate (6 mg/day) and paracetamol (>4 g/day) at his peril. He denied taking any other drugs on that occasion. Because his clinical condition had not improved, he went to the hospital, where he underwent medical tests such as complete blood count (CBC): hemoglobin 13.2 g/dL, hematocrit 39.6%, platelets 343,000/mm^3^, white blood cells (leucocytes 5200/mm^3^), liver function Aspartate transaminase (AST) 39.8 U/L, Alanine transaminase (ALT) 40.1 U/L, gamma-glutamyl transferase (GGT) 346 U/L, and alkaline phosphatase 98.5 U/L. In addition, the NS1 test for dengue was negative and the abdominal ultrasound showed the liver with diffuse increase in parenchymal echogenicity, indicating mild/moderate steatosis. The patient was diagnosed with hepatitis due to the clinical signs assessed, results of laboratory tests, and excessive intake of paracetamol. The patient was hospitalized, and after 4 days of intravenous hydration and clinical assessment, he was discharged from the hospital.

Fifteen days after his discharge, the patient related that he had daily high fever, appetite loss, sweating, weight loss (8 kg), and progressive worsening. On August 6, 2016, the infectious disease specialist ordered new medical tests when examining the patient. The results revealed that blood count was hemoglobin 10.2 g/dL; hematocrit 30.7%; red blood cells 4.16 million/mm^3^; and platelets 200,000/mm^3^. It also revealed anisocytosis, microcytosis, and hypochromia; white blood cells were leucocytes 9700/mm^3^, banded neutrophils 6%, segmented neutrophils 76%, and lymphocytes 10%. Liver function tests indicated aspartate transaminase (AST) 64 U/L, alanine transaminase (ALT) 85 U/L, gamma-glutamyl transpeptidase (GGT) 560 U/L, and lactic dehydrogenase 248 U/L. C-reactive protein was 134.29 mg/L. Serologies were ordered for leptospirosis, rickettsialpox, hepatitis, syphilis, Zika virus, Chikungunya, Epstein-Barr virus, cytomegalovirus, toxoplasmosis, and human immunodeficiency virus (HIV). All of them had negative results.

Furthermore, blood cultures were conventionally performed using the Bact/Alert 3D 120 automated microbial detection system. The initial incubation was performed with chocolate agar medium, in 5–10% CO_2_ at 35°C–37°C for 24 hours. Three blood samples were collected in the first 24 hours, at a minimum 15-minute interval between them, using venous puncture in different places. The colonies isolated on chocolate agar plates were identified by colonial morphology, gram stain, and oxidase reaction. Colonies appeared smooth, round, uniform, grey/brown, and 1 mm in diameter. The oxidase test and tributyrin agar test were positive. This result was positive for the presence of the microorganism *M. osloensis* in the three samples.

Transesophageal echocardiography (TEE) was ordered, and it showed prolapsed mitral valve involving the posterior leaflet (P2 segment) with an echogenic structure adhered to the atrial face, with approximate dimensions of 0.47 × 0.85 cm, compatible with endocardial vegetation, determining failure of coaptation and a moderate degree of valvular insufficiency and eccentric jet. Intracavitary thrombi were absent, and systolic and diastolic functions of the left ventricle were normal. According to the criteria of Habib et al. [8], the diagnosis of IE was confirmed, and the patient was hospitalized for intravenous antibiotic therapy.

Antibiotic therapy was prescribed from August 8 to September 28 with the administration of vancomycin for four days, gentamicin for 10 days, and ampicillin for 6 days, and after the result of blood culture, ceftriaxone was introduced for 36 days. Remission of signs and symptoms was observed as of August 12. Laboratory tests results were better, and blood cultures were sterile. Theses exams were repeated on August 21 and on September 15. Their results showed no alteration.

After completing the therapeutic treatment with specific antibiotics, the patient underwent serial laboratory tests and was hospitalized. Another TEE was carried (09/21), and it revealed chordal rupture and moderate mitral regurgitation. Surgical correction was performed.

On September 26, the patient underwent implantation of a mitral valve prosthesis. Surgery was performed without any complications. However, neither the culture nor the pathological anatomy of the surgically removed valve was performed. On October 06, the patient was discharged from the hospital with a prescription for oral anticoagulant warfarin.

Eighteen months after the endocarditis episode, the patient was asymptomatic, in good physical condition. He attends follow-up visits with a cardiologist at the same hospital.

## 3. Discussion

The diagnosis of IE is challenging because of its quite variable clinical history, the epidemiological profile in evolution, the presence or absence of preexisting heart disease, prosthetic valves, and/or implantable cardiac devices, as well as its acute, subacute, or chronic nature [8]. Other diagnostic hypotheses can be considered such as other site infections, rheumatic disease, tumors, autoimmune diseases, and neurological diseases such as meningitis [9]. However, early evaluation by a cardiologist may help to improve diagnosis accuracy or avoid delays in final diagnosis [8, 10, 11].

Fever with chills, lack of appetite, and weight loss are found in up to 90% of IE cases, heart murmurs in up to 85%, and embolic complications around 25% at the time of diagnosis [8–10]. Concomitant fever and embolic phenomena strongly lead to the diagnosis of IE [10]. The case description showed these unspecific symptoms (fever, myalgia, anorexia, and weight loss) similar to flu status. The presence of these signs and symptoms despite being common in IE can induce other diagnostic hypotheses. Microbiological investigations, such as blood culture, laboratory tests, biomarkers and mainly imaging, TEE and transthoracic, should be evaluated when suspecting IE, with three echocardiographic findings being the main criteria in the diagnosis of IE as vegetation, abscess or pseudoaneurysm, and new dehiscence of a prosthetic valve [8, 11].

The first case report of IE caused by the microorganism *M. osloensis* dates from 1982, and it was described by Stryker et al. [12]. This study would be the fourth case since two more recent episodes were described by Gagnard et al. [2] in 2015, featuring individuals over 50 years old and significant immune impairment. The first manifested chronic lymphocytic leukemia and the second had a previous history of two episodes of IE in mitral valve prosthesis by *Streptococcus* and *Staphylococcus* sp., Hodgkin's lymphoma, and renal transplantation.

The patient of this case, affected by endocarditis due to *M. osloensis* infection, is different from those previously described in the literature, because he was a young adult, under 50 years old, immunocompetent, with no comorbidities and no obvious focus of infection. The scientific literature revealed less than 40 reports of infections by this species [2, 5], and most patients were at the extremes of age, under 6 and over 50 years old, and had a history of hematological malignancies [2, 13], solid neoplasms [14, 15], deficiency of complement [16], osteomyelitis [17], and previous surgeries [2], that is, documented presence of some factor predisposing vulnerability of the immune system [2, 11]. Probably, our patient had a viral infection of the upper airways. Then, this could have caused a drop in the immune status with an increased susceptibility to bacteremia. Therefore, this case highlights a rare impairment of the endocardium of an immunocompetent adult, with no comorbidities and no focus of infection [8, 9, 11].

Furthermore, this case emphasizes that the diagnosis of IE must be considered even in patients with nonspecific symptoms and unexpected epidemiological profile. Although endocarditis presents a variable prognosis, mostly good, it is a serious infection that can lead to death.
